# Kutane Pseudolymphome nach Hirudotherapie: Fallbericht und Literaturübersicht

**DOI:** 10.1007/s00105-021-04812-6

**Published:** 2021-04-21

**Authors:** Susanne Hanner, Hannah Stroh, Alexander Enk, Jochen Hoffmann

**Affiliations:** 1 Hautklinik, Universität Heidelberg, Im Neuenheimer Feld 440, 69120 Heidelberg, Deutschland; 2 Haut- und Laserzentrum Heidelberg, Dres. Durani, Bergheimer Str. 56a, 69115 Heidelberg, Deutschland

**Keywords:** Blutegeltherapie, Lymphoproliferation, Histologie, Immunhistochemie, Molekularpathologie, Medicinal leech therapy, Lymphoproliferation, Histology, Immunochemistry, Molecular pathology

## Abstract

Unter dem Begriff Pseudolymphom (PSL) versteht man eine benigne, reaktive Lymphoproliferation der Haut, die klinisch und/oder histologisch ein malignes Lymphom simulieren kann. Die genaue Ätiopathogenese ist bis heute nicht gänzlich geklärt. Man unterscheidet die primären, idiopathischen PSL ohne erkennbare Ursache von den sekundären PSL mit bekanntem Stimulus. Wir berichten über das Auftreten von Pseudolymphomen nach einer Behandlung mit medizinischen Blutegeln (Hirudotherapie). Bisher wurden nach bestem Wissen und Gewissen insgesamt nur 9 Fälle von kutanen PSL nach Hirudotherapie in der Literatur beschrieben.

## Abstract (en)

The term cutaneous pseudolymphoma (C-PSL) is defined in the literature as a benign, reactive lymphoproliferation that clinically and/or histopathologically imitates cutaneous lymphoma. The exact etiopathogenesis has not been fully elucidated to date. A distinction is made between primary, idiopathic PSL without an identifiable cause and secondary PSL with a known stimulus. We report the occurrence of pseudolymphoma after treatment with medicinal leeches (hirudotherapy). To the best of our knowledge, a total of only nine cases of cutaneous PSL after hirudotherapy have been reported in the literature to date.

## Anamnese

Eine 76-jährige Patientin stellte sich mit seit über 1 Jahr bestehenden, juckenden, lividen bis erythematösen Plaques an der rechten Ferse sowie im Bereich des unteren Rückens in unserer allgemeinen Ambulanz vor. Auf intensive Nachfrage berichtete die Patientin, dass sie sich aufgrund von Gelenkschmerzen im Lendenwirbelsäulenbereich und eines Fersensporns einige Wochen vor Auftreten der Effloreszenzen 2 Sitzungen einer Blutegeltherapie durch eine Heilpraktikerin unterzogen hatte. Es wurden keine relevanten Nebenerkrankungen angegeben. Die Dauermedikation bestand aus Metoprolol, Ramipril sowie L‑Thyroxin.

## Diagnostik

### Hautbefund

**Figure 1:**
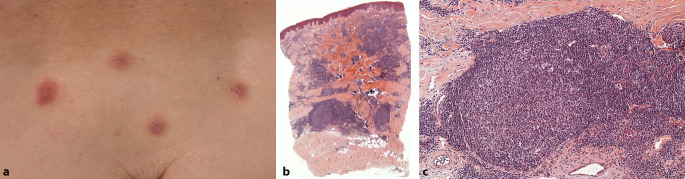

Bei der Ganzkörperinspektion zeigten sich an oben genannten Ansatzstellen der Blutegel insgesamt 7 livide bis erythematöse, infiltrierte Plaques (Abb. 1a). Das übrige Integument und die Schleimhäute kamen unauffällig zur Darstellung. Zur weiteren diagnostischen Einordnung erfolgte die Durchführung einer Probebiopsie.

### Histopathologischer Befund

Es zeigten sich knotige, lymphozytär dominierte Entzündungsinfiltrate mit vereinzelten eosinophilen Granulozyten und Plasmazellen um eine zentral gelegene, zur Epidermis senkrecht stehende Narbenzone. Zur Tiefe hin waren regelrecht ausgebildete, reaktive Follikelzentren mit Kerntrümmermakrophagen, zahlreichen Mitosen und erhaltener Mantelzone erkennbar (Abb. 1b, c).

### Immunhistochemischer Befund

Immunhistochemische Färbungen zeigten ein gemischtzelliges Infiltrat mit Nachweis von zahlreichen kleinen B‑ und T‑Lymphozyten (Positivität für CD4- und CD20).

### Laborbefund

Die Lues- und Borrelienserologie waren negativ.

## Diagnose

In Zusammenschau der Anamnese, des klinischen Befundes, der Histopathologie sowie der Immunhistochemie stellten wir die Diagnose kutaner Pseudolymphome (PSL) nach medizinischer Blutegeltherapie.

## Therapie und Verlauf

Es wurde eine topische Behandlung mit Mometason eingeleitet. Bei der letzten Wiedervorstellung zur Verlaufskontrolle nach 24 Monaten zeigten sich nur noch dezent sichtbare livide Verfärbungen im Bereich der ehemaligen Knoten an der Lendenwirbelsäule.

## Diskussion

Unter dem Begriff Pseudolymphom (PSL) oder kutane lymphoide Hyperplasie versteht man eine benigne, reaktive Lymphoproliferation der Haut, die klinisch und/oder histologisch ein malignes Lymphom simulieren kann [1, 2]. Es handelt sich um eine heterogene Gruppe von Entitäten, die sich klinisch, histologisch, immunhistochemisch sowie ätiologisch unterscheiden [1]. In den letzten Jahren wurden in der Literatur zahlreiche Klassifikationen für Pseudolymphome publiziert [1]. Diese beinhalten Einteilungen der PSL nach dem Immunphänotyp (T-Zell‑, B‑Zell- oder gemischtzellig), den histopathologischen Merkmalen, der Ätiologie sowie nach unterschiedlichen klinischen Merkmalen [2]. Eine international etablierte, konsensusbasierte Klassifikation analog der WHO(World Health Organization)-EORTC(European Organization for Research and Treatment of Cancer)-Klassifikation für kutane Lymphome liegt bis zum jetzigen Zeitpunkt nicht vor [1]. Man unterscheidet die primären, idiopathischen PSL ohne erkennbare Ursache von den sekundären PSL mit bekanntem Stimulus [3, 4]. Triggerfaktoren umfassen u. a. verschiedene Medikamente und Infektionen, aber auch Traumata wie Tattoos, Insektenstiche, Akupunktur und Impfungen [2, 4–6]. Ein typisches histologisches Merkmal der kutanen PSL ist das Vorhandensein eines polyklonalen, lymphozytären Infiltrats in der oberen Dermis [4]. Charakteristisch für die PSL ist ein gutartiger Verlauf. Aufgrund der selten beschriebenen malignen Transformation sowie der mitunter erheblichen differenzialdiagnostischen Schwierigkeiten sollten regelmäßige Nachsorgeuntersuchungen erfolgen [1, 5].

In dem vorliegenden Fall kam es zu einem Auftreten von Pseudolymphomen nach einer Behandlung mit medizinischen Blutegeln (Hirudotherapie). Bisher wurden nach bestem Wissen und Gewissen insgesamt nur 9 Fälle von kutanen PSL nach Hirudotherapie in der Literatur beschrieben [3–11], die in Tab. 1 zusammengefasst sind. Histologisch und immunhistochemisch zeigte sich ein breites Bild ohne Dominanz eines Musters. Therapeutisch wurde zumeist entweder eine topische oder intraläsionale Steroidbehandlung durchgeführt. Hierunter kam es in den berichteten Fällen zu einer Befundbesserung.

**Table 1:** 

Autor (Jahr, Referenz)	Alter/Geschlecht	Lokalisation	Indikation der Hirudotherapie	Immunhistochemie	Therapie	Follow-up
Hanner et al. (aktueller Fall)	76/w	Unterer Rücken (lumbal), Ferse	Gelenkschmerzen, Fersensporn	Gemischt T‑ und B‑lymphozytäres Infiltrat	Topische ST	Langsam rückläufige Läsion
Sepaskhah et al. (2020), [11]	44/w	Schienbein	Erythema nodosum	Nicht durchgeführt	Systemische ST	Rückläufige Läsionen
					Topische ST	
					Intraläsionale ST	
Sadati et al. (2019), [9]	45/w	Untere Extremitäten	Varikose und Schmerzen	Nicht durchgeführt	Topische ST	Komplett rückläufige Läsionen
					Kryotherapie	
Temiz et al. (2019), [5]	54/m	Hals	Unbekannt	T‑lymphozytär dominiertes Infiltrat	Intraläsionale ST	Nach 1 Monat: rückläufige Läsionen
						Nach 6 Monaten: kein Rezidiv
Aktas et al. (2018), [10]	65/w	Unterer Rücken (lumbal)	Rückenschmerzen	Nicht durchgeführt	Intraläsionale ST	Rückläufige Läsionen
					Kryotherapie	
Tupikowska et al. (2018), [4]	38/w	Regio pubica	Uterusmyom	Gemischtzelliges, T‑lymphozytär dominiertes Infiltrat	Orale AH	Langsam rückläufige Läsionen
					Topische ST	
					Intraläsionale ST	
					Intramuskuläre ST	
					Kryotherapie	
Altamura et al. (2014), [7]	50er/w	Rücken	Fibromyalgie	B‑lymphozytär dominiertes Infiltrat	Topische ST	Nach 1 Monat: komplett rückläufige Läsionen
						Nach 15 Monaten: kein Rezidiv
Khelifa et al. (2013), [6]	77/w	Unterer Rücken (lumbal)	Rückenschmerzen bei lumbaler Spinalkanalstenose	Gemischt T‑ und B‑lymphozytäres Infiltrat	Topische und intraläsionale ST	Rückläufige Läsionen
Choi et al. (2012), [3]	52/m	Unterlid bds.	Infraorbitale Augenringe	Gemischtzelliges, T‑lymphozytär dominiertes Infiltrat	Intraläsionale ST	Nach 3 Monaten: rückläufige Läsionen
Smolle et al. (2000), [8]	56/w	Unterschenkel bds.	Chronisch venöse Insuffizienz	B‑lymphozytär dominiertes Infiltrat	Intraläsionale ST	Langsam rückläufige Läsionen

*w* weiblich, *m* männlich, *ST* Steroidtherapie, *AH* Antihistaminika, *bds*. beidseits

Im letzten Jahrzehnt gewann die Blutegeltherapie mit neuen Einsatzbereichen an zunehmender Bedeutung [8]. Die Hauptindikationen der medizinischen Blutegeltherapie stellen unterschiedliche Gelenkerkrankungen wie Osteoarthritis und Epikondylitis, Venenerkrankungen sowie die Lappenplastik der plastischen Chirurgie dar [12]. Die Blutegel umfassen mehr als 600 verschiedene Arten, der bekannteste und in der Medizin vorrangig eingesetzte Vertreter ist der *Hirudo medicinalis* [6, 12]. Bis dato konnten mehr als 20 bioaktive Substanzen im Speichelsekret der Blutegel mit u. a. analgetischen, antiinflammatorischen, plättchenhemmenden, gerinnungshemmenden und Thrombin-regulatorischen Funktionen sowie mit antimikrobiellen und extrazellulären Matrix-abbauenden Wirkungen identifiziert werden [12]. Mögliche Komplikationen sind Infektionen, Blutungen, Anämie, allergische Reaktionen sowie Narbenbildung [3]. Zudem können – wie im dargelegten Fall – kutane PSL als seltene, aber beachtenswerte Nebenwirkungen der Blutegeltherapie auftreten. Die Pathogenese der kutanen PSL nach Hirudotherapie ist bis heute nicht gänzlich geklärt. Die pseudolymphomatösen Reaktionen werden vermutlich durch den Biss der scharfen Zähne, durch Substanzen der Blutegel oder durch infektiöse Erreger, die während der Behandlung übertragen werden, bedingt [6, 8]. Als möglicher Pathomechanismus wird eine verzögerte Hypersensitivitätsreaktion auf Bestandteile der Insekten diskutiert [3]. In der Literatur wurden selten sowohl irritative als auch allergische Kontaktdermatitiden nach medizinischer Blutegeltherapie beschrieben [13]. Hirudin kommt als ein mögliches, auslösendes Agens in Betracht [13]. Die definitive Identifikation des auslösenden Agens ist bei mehr als 100 nachgewiesenen Proteinen mit einem molekularen Gewicht zwischen 10 und 97 kD im Speichelsekret der Blutegel allerdings schwierig [13].

Auch für erfahrene Mediziner ist die Diagnose eines Pseudolymphoms oftmals eine Herausforderung und erfordert nicht selten die Korrelation aller Befunde – der Klinik, der Histologie, der Immunhistochemie und der Molekularpathologie. Es ist zu erwarten, dass mit zunehmender Anwendung der Blutegeltherapie in unterschiedlichen Bereichen der Medizin auch die kutanen Nebenwirkungen häufiger beobachtet werden. Daher sollte bei der differenzialdiagnostischen Abklärung von Pseudolymphomen auch diese seltene Ursache bedacht werden.

## Fazit für die Praxis

- Auch für erfahrene Mediziner ist die Diagnose eines Pseudolymphoms oftmals eine Herausforderung und erfordert nicht selten die Korrelation aller Befunde – der Klinik, der Histologie, der Immunhistochemie und der Molekularpathologie.
- Es ist zu erwarten, dass mit zunehmender Anwendung der Blutegeltherapie in unterschiedlichen Bereichen der Medizin auch die kutanen Nebenwirkungen häufiger beobachtet werden. Daher sollte bei der differenzialdiagnostischen Abklärung von Pseudolymphomen auch diese seltene Ursache bedacht werden.
